# Single Fixed-Time Post-Cervical Insemination in Gilts with Buserelin

**DOI:** 10.3390/ani11061567

**Published:** 2021-05-27

**Authors:** Andrés Suárez-Usbeck, Olga Mitjana, María Teresa Tejedor, Cristina Bonastre, Jorge Sistac, Antonio Ubiergo, María Victoria Falceto

**Affiliations:** 1 Department of Animal Pathology, Agroalimentary Institute of Aragon-IA2, University of Zaragoza-CITA, C/Miguel Servet 177, 50013 Zaragoza, Spain; mvzandressuarez@gmail.com (A.S.-U.); omitjana@unizar.es (O.M.); cbonastr@unizar.es (C.B.); vfalceto@unizar.es (M.V.F.); 2 Department of Anatomy, Embryology and Animal Genetics, Genetic s Area, Faculty of Veterinary Medicine, University of Zaragoza, C/Miguel Servet 177, 50013 Zaragoza, Spain; 3 CIBER CV (University of Zaragoza—IIS), Faculty of Veterinary Medicine, University of Zaragoza, C/Miguel Servet 177, 50013 Zaragoza, Spain; 4 Granja Fabardo (Mazana Grupo Empresarial), 22480 Capella, Huesca, Spain; jorge.sistac@mazana.es; 5 Semen Costean, CIA, 22312 Costean, Huesca, Spain; aubiergovet@yahoo.es

**Keywords:** fixed-time insemination, buserelin, gilt, post-cervical

## Abstract

**Simple Summary:**

Current protocols for gilts recommend the deposit of two/three semen doses (2–4 × 10^9^ sperm/dose) by cervical artificial insemination (CAI), 12–24 h after estrus detection. If ovulation were predictable, gilts could be bred only once using fixed-time artificial insemination (FTAI). Using a specific catheter makes the postcervical deposition of semen possible (PCAI). This work explored the use of combining FTAI-PCAI with buserelin in gilts. In the control group (C; *n* = 240), gilts were inseminated twice (8 and 12 h from estrus onset). Gilts in the treatment group (T; *n* = 226) received buserelin (10 μg, intramuscular) 120 h after altrenogest treatment (18 d) and one single PCAI 30–33 h after buserelin administration. No significant differences were found in reproductive and production performance between groups (*p* > 0.05). Piglets’ birth weight was greater in the T group (*p* < 0.001). Estrus duration was significantly shorter in the T group (*p* < 0.001). Delivery batch length significantly differed depending on the season (*p* < 0.05); both groups only differed significantly in spring (*p* = 0.018), with a shorter duration in the T group. This new FTAI-PCAI protocol with buserelin is recommended in gilts, helping with optimization of genetic diffusion, boars, and semen doses.

**Abstract:**

Current protocols for gilts recommend the deposit of multiple semen doses in the cervix each 12–24 h after estrus detection. Our objectives were: (1) to determine the effect of buserelin and a single fixed-time artificial insemination using the new post-cervical artificial insemination technique (FTAI-PCAI) on reproductive and productive performance in gilts, and (2) to compare this protocol with conventional estrus detection and double PCAI without hormonal induction. In the control group (C; *n* = 240), gilts were inseminated twice (8 and 12 h from estrus onset). Gilts in the treatment group (T; *n* = 226) received buserelin (10 μg, intramuscular) 120 h after altrenogest treatment (18 d) and one single PCAI 30–33 h after buserelin administration. The groups did not differ in reproductive and production performance (*p* > 0.05). The T group showed greater piglet birth weight and shorter estrus duration (*p* < 0.001). Delivery batch length differed significantly depending on the season (*p* < 0.05); the shortest length corresponded to autumn. Both groups only differed significantly in spring (*p* = 0.018), with a shorter length in the T group. This new FTAI-PCAI protocol with buserelin is recommended in gilts, helping with optimization of genetic diffusion, boars, and semen doses.

## 1. Introduction

The main aim of artificial insemination (AI) is to deposit enough viable sperm in the appropriate place of the female genital tract at the optimal moment relative to ovulation. For gilts, current protocols recommend the deposit of multiple semen doses (two or three) in the cervix each 12–24 h after estrus detection (2–4 × 10^9^ sperm at 60–100 mL per dose, stored at 17 °C for a maximum period of 3–7 days, depending on the extender) [1,2]. Reasons for repeated AI lie in the brief viability of both oocytes and spermatozoa in the gilt reproductive tract and the difficultly of exactly predicting ovulation during estrus [3].

The post-cervical artificial insemination (PCAI) procedure was proposed as a new technique for depositing semen in the uterine body, as an alternative to cervical AI (CAI). PCAI and CAI differ not only in the semen deposition site but also in the sperm concentration and dose volume used for AI [2,4,5]. PCAI promotes efficient genetic progress, minimizes semen backflow during the insemination process, and decreases the time to conduct the AI procedure, without reduction in litter size and farrowing rate [2,4,6,7]. The suitability of PCAI in gilts has been previously demonstrated [2,8].

Fixed-time artificial insemination (FTAI) involves one single semen dose applied within a period of 0–24 h before ovulation [9]. A better understanding of regulation mechanisms for follicular development and ovulation has enabled new perspectives on ovulation control in gilts, and therefore for FTAI development [10]. In gilts, both estrus duration and the estrus onset-ovulation interval are highly variable; therefore, controlling ovulation timing is needed [1,9,11].

Gonadotropin-releasing hormone (GnRH) analogues, luteinizing hormone (LH), and human chorionic gonadotropin (hCG) have been efficiently used to induce ovulation in weaned sows and gilts, always with CAI [1]. Several studies have recommended the combined use of porcine LH (pLH) plus equine chorionic gonadotropin (eCG) with single or double PCAI in sows [12,13]. Additionally, one single pLH application at the estrus onset has shown good results in sows with either single [14] or double PCAI [15,16]. Several GnRH agonists have been used as ovulation inductors, as a previous step to various AI techniques. Licerelin with CAI has been applied to sows [17]. Buserelin with PCAI has been used in sows [18], while buserelin with double CAI has been applied in gilts [19]. In both sows and gilts, goserelin with CAI has been used [11]. Triptorelin with PCAI has been used in sows [20,21], and more recently, a single-FTAI triptorelin protocol with CAI was used in gilts [22].

Furthermore, good reproductive results were obtained in gilts with an altrenogest treatment followed at 115–120 h after completion by buserelin administration, which enabled FTAI-CAI to be performed 30–33 h later [19]. The simplest protocols with single hormonal application showed effectiveness in synchronizing ovulation and reducing the number of inseminations [20,23]. This reduction in cost and labour of hormonal protocols could ease single FTAI use in routine AI protocols in gilts. Rodrigues [24] compared two different protocols to synchronize ovulation before FTAI-CAI (eCG vs. Triptorelin acetate) in gilts from an experimental farm; these protocols were based on procedures previously used in multiparous sows, and farrowing rates and litter sizes were lower than those obtained with the current AI technique. Therefore, more research is needed for the establishment of new protocols to improve reproductive performance in gilts, considering the differences between farms. 

In this line of thought, the objective of this work was twofold. The first objective of the present study was to determine the effect of buserelin (as a synchronization protocol) and a single FTAI (using the new PCAI technique) on reproductive and productive performance when used in gilts from a commercial farm. The second objective was to compare the effects of this protocol with those of conventional estrus detection and double PCAI without hormonal induction on reproductive and productive performance in these gilts. 

## 2. Materials and Methods 

### 2.1. Ethical Declaration 

This study complied with the ARRIVE guidelines [25], Council Directive 2008/120/EC outlining minimum standards for the protection of pigs, and Directive 2010/63/EU of the European Parliament and of the Council of 22 September 2010 on the protection of animals used for scientific purposes. All procedures in these experiments were conducted consistently with the precepts of animal welfare and approved by the Committee of Ethics in Animal Experimentation of Universidad de Zaragoza (protocol No. PI35/21NE). 

### 2.2. Animals

This study was conducted according to the Spanish standard commercial swine production on a breed farm located near Huesca (Capella, north-eastern Spain). A total of 482 gilts were used in the study; these gilts were 255–270 days old, weighed 150 ± 5 kg (SD), and had two previously detected periods of estrus. All animals belonged to a hyper-prolific genetic line (DanBred, DANBRED P/S, Herlev, Denmark).

Twice a day, gilts were fed a commercial diet (3 kg/day) containing 3200 kcal EM/kg, 14% PB, and 0.7% digestible lysine. All gilts were treated with altrenogest (REGUMATE^®^, Merck & Co., Inc., Kenilworth, NJ, USA) administered orally and individually for 18 days. Water was available ad libitum. After AI, the gilts were housed in individual pens (0.65 × 2 m) until pregnancy status diagnosis occurred (24 days after AI).

Semen doses used in this study were obtained from 49 different Pietrain boars, belonging to an insemination center, (Semen Costean; CIA, Costean, Spain), located several Km far from the farm where the gilts were housed. Boars received a specific diet; 2.6–3.0 kg for pig males between 200 and 300 kg, containing 3000 kcal EM/kg and 0.5% digestive lysine. 

### 2.3. Sperm Collection

Semen doses used in this study were obtained once a week using the gloved-hand technique for collections and then filtered to remove the gel. The average number of spermatozoa was assessed using a BRAND^®^ counting chamber BLAUBRAND^®^ Bürker pattern (Merck & Co., Inc., Kenilworth, NJ, USA). Spermatozoa motility, agglutination, and abnormalities were analyzed using the AndroVision^®^ software 12500/0000 (Minitube, Tiefenbach, Germany). According to current protocols at the insemination center, we were only provided with ejaculates complying the minimum requirements (motility > 80% and total abnormalities < 20%). Immediately after evaluation, each ejaculate was fully diluted in a commercial extender at 37 °C (VITASEM^®^, Magapor, Ejea de los Caballeros, Spain). The ejaculates were combined in heterospermic doses and stored in bag doses containing 1.5 × 10^9^ spermatozoa per 60 mL for PCAI. These heterospermic doses were identical in each insemination batch for both groups considered in this study. Doses were stored at 15 to 18 °C for 72 h. 

### 2.4. Oestrus Detection and Duration

After Regumate treatment, gilts were randomly assigned to control (C; *n* = 244) and treatment (T; *n* = 238) groups. Estrus detection was performed twice daily (8:00 and 13:00) in both groups, using mature boars and the standing reflex in response to back pressure, as well as the presence of reddening and swelling of the vulva, according to the sequence characterized by Signoret [26]. Once estrus was detected in a gilt, the sign + was marked on its back, and the day and time were recorded. The estrus duration was the interval (h) from the establishment to the stop of the standing reflex. When no estrus signs were present, gilts were considered in anestrus and removed from the experiment.

### 2.5. Insemination Assays

In the C group, 240 gilts were inseminated for the first time 8 h after estrus onset and again 12 h later, during the estrus period. Both inseminations were carried out by means of the PCAI technique. 

Gilts in the T group received 10 µg of intramuscular buserelin (Porceptal^®^, MSD, Salamanca, Spain) 120 h after the last altrenogest dose. Estrus detection was performed 30–33 h later. Only gilts with standing reflex and reddening and swelling of the vulva were prepared for single-dose PCAI; 226 gilts were inseminated in the T group. 

Gilts in C group were inseminated between 8 and 8:30 and between 13:30 and 14:00; inseminations of gilts in T group were carried out between 13: 00 and 14:00. Semen doses were obtained less than 24 h before use for both first insemination of C group and unique insemination of T group, and therefore they have been obtained less than 48 h before its use in second insemination of C group. During transport and in the farm, semen doses were kept at 17 °C.

**Figure 1:**
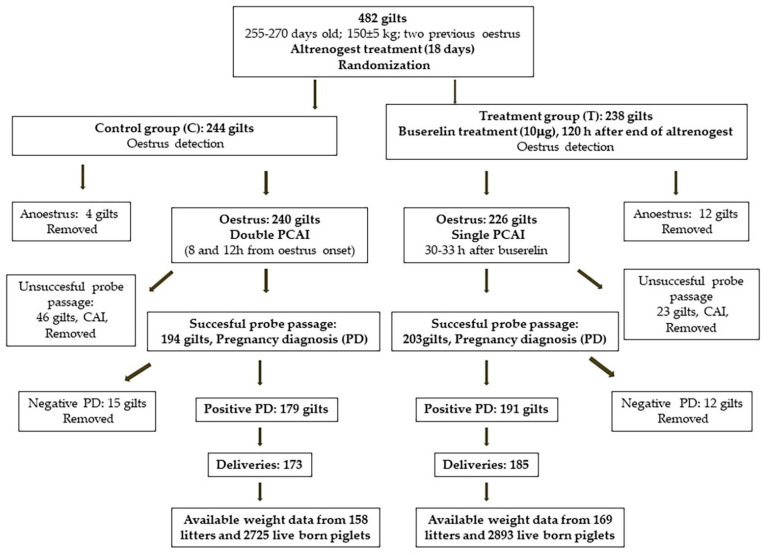
Study design. PCAI: post cervical artificial insemination; CAI: cervical artificial insemination.

All inseminations were carried out between December 2019 and August 2020 (eight batches for each group). In both the C and T groups, PCAI was conducted in the same way; without the presence of a boar, we used a specific PCAI probe for gilts (MAGAPLUS N^®^, Magapor, Ejea de los Caballeros, Spain) and, as a guide, the foam tip catheter for gilts that was manufactured by Magapor (Ejea de los Caballeros, Spain). Dose concentration was always 1.5 × 10^9^ spermatozoa per 60 mL. When the PCAI probe could not pass through the cervix (unsuccessful probe passage), gilts in either of the two groups were submitted to CAI and removed from the study; from then on, only 194 and 203 gilts were considered for the C and T groups, respectively. Occurrences of AI problems such as difficult probe passage, bleeding, semen backflow, and metritis were individually recorded. Figure 1 shows the study design.

### 2.6. Pregnancy Detection and Return to Oestrus

Trans-abdominal ultrasonography (Future-1^®^, Inserbo, Spain) 28 days after insemination was used for pregnancy diagnosis. The pregnancy rate was calculated as the proportion of inseminated females that were pregnant. After positive diagnosis, the AI technicians recorded returns to estrus, abortions, and deaths. Reaction to boar stimulation or back pressure evidenced the return to estrus. 

### 2.7. Farrowing and Litter Parameters 

The farrowing rate was calculated as the proportion of inseminated females that farrowed. Additionally, the frequencies of dystocia, total piglets born/litter, live-born piglets/litter, stillborn piglets/litter, and mummies/litter were recorded. Gestation duration and weaning-estrus (days) were recorded for every farrowing gilt. The delivery batch duration was measured as the period (days) between the first and last deliveries in the batch. 

Live-born piglets were individually weighed (kg) within 24 h after birth and before cross-fostering. In this operation, a calibrated scale was used (ECE 50K-2N, Kern & Sohn GmbH, Balingen, Germany). Litter weight was also estimated (kg). 

### 2.8. Statistical Analysis

All statistical analyses were performed using SPSS v. 26. Categorical variables were analyzed by cross-tabulation, and percentages were compared using Pearson’s χ^2^ test. Quantitative variables were summarized as mean ± standard error (SE). Coefficient of variation (CV = standard deviation/mean) was calculated for every litter, as an estimation of litter homogeneity. Summaries for time variables (durations and intervals) also included median ± SE.

ANOVA was used to compare the C and T groups (fixed effects) for total piglets born/litter. The ANOVA model for live-born piglets/litter and individual and litter weight within 24 h after birth and before cross-fostering also included total piglets born/litter as a covariate. A non-parametric test (Mann–Whitney U test) was used to compare the C and T groups for stillborn piglets/litter, mummies/litter, and CV. Kaplan Meier’s survival analysis was applied to time variables: Breslow’s test was used for comparing the C and T groups. Differences were considered significant at *p* < 0.05.

## 3. Results

Table 1 shows the AI results for every inseminated gilt (240 and 226 individuals in the C and T groups, respectively). Probe passage was never forced. Difficult passage means that difficulties occurred but finally the probe managed to gently pass through the cervix. Differently, unsuccessful probe passage means probe passage was impossible. As stated in the Material and Methods section, gilts in the C group were inseminated twice; unsuccessful probe passage in one of the AIs was enough for a gilt to be removed from the study. The C and T groups significantly differed in the proportion of gilts showing successful probe passage (194/240: 80.8% vs. 203/226: 89.8% in the C and T groups, respectively, *p* = 0.006). Frequencies for other problems (difficult probe passage, bleeding, semen backflow, metritis) did not differ significantly between groups in the animals that remained in the study (42/194: 21.9% and 30/203: 14.8% in the C and T groups, respectively).

Table 2 presents data regarding the reproductive and production performance of gilts in both the C and T groups. 

Data regarding estrus, gestation, and weaning-estrus are shown in Table 3. 

Table 4 presents data regarding the delivery batch duration of considered gilts. Deliveries occurred in eight different batches throughout 2020: three batches in spring (21 March–20 June), one batch in summer (21 June–20 September), and five batches in autumn (21 September–20 December). 

Delivery batch length differed significantly depending on the season; in summer, duration was longer than in autumn (*p* < 0.001) and spring (*p* = 0.006), and a significant difference (*p* = 0.020) was also found for spring versus autumn. According to median values, 50% of gilts gave birth in the two first days of the delivery batch in autumn, but this accumulate percentage was not reached until the third day in spring and the fourth day in summer. When the C and T groups were compared within season, they only differed significantly in spring (*p* = 0.018), with a shorter duration in the T group; a difference of one day was observed in the median value, in favor of the T group.

Table 5 indicates piglets’ weight within 24 h after birth and before cross-fostering (individual and litter weight, CV). 

Significantly greater values for individual weight were found for the T group *(p* < 0.001), but no significant differences were found for litter weight (*p* > 0.050). Low CV values were found for both the C and T groups. No significant differences for CV were detected (*p* = 0.092); however, both 25th and 50th percentiles were lower in the T group (1.45 and 5.77, respectively) than in the C group (3.55 and 7.27, respectively), pointing to a tendency for more homogeneous litters in the T group.

## 4. Discussion

The use of PCAI has increased worldwide. PCAI has been widely studied in multiparous and primiparous sows [27,28,29,30,31,32,33,34,35,36]. In recent years, there have been many PCAI trials in nulliparous gilts [2,8,31,37,38,39], with different results. Therefore, more studies and research are needed to improve the PCAI technique in gilts. To the best of our knowledge, this is the first study about using PCAI in a single-dose FTAI for gilts. 

The main objective of applying the PCAI technique in gilts is the successful insertion of a cannula through the cervix, hampered by the small size of the reproductive tract. In the present study, the success of the probe passage through the cervix reached 80.8% and 89.8% for the C and T groups, respectively (*p* < 0.05), in contradiction with previous studies that advised against PCAI for gilts [8,31,37]. However, these results were similar to those obtained by other researchers [38,39], including a previous work of ours, where we recommended PCAI for Landrace × Large White gilts without compromising reproductive parameters’ efficiency [2]. The frequencies of problems during AI (difficult probe passage, semen backflow, metritis, and bleeding) did not differ significantly between groups (C: 21.9% and T: 14.8%; *p* > 0.05). These results suggest that the use of a specific probe by a qualified technician did not result in severe lesions in the cervix of gilts, confirming previous reports [40]. Post-cervical insemination provides a number of advantages, such as a reduced sperm number requirement, and therefore, it can be used in new FTAI protocols to accelerate genetic improvement programs [7]. On the other hand, although PCAI was used in both groups, each gilt was inseminated twice in the C group and only once in the T group; therefore, the probability of unsuccessful probe passage decreased in the T group, as expected.

Several analogues of GnRH have been used in sows and gilts as ovulation inductors, and their effects on reproductive parameters have been studied. D-Phe6-LHRH, a luteinizing hormone-releasing hormone antagonist, is a GnRH + hCG analogue (Gonavet^®^, Berlin-Chemie, Berlin, Germany) that produced variable reproductive results in gilts and sows [41]. A trial using eCG (Folligon^®^, Intervet, Whitby, ON, Canada) and double FTAI protocols only evaluated LH, FSH levels, ovulation rate, and quality in gilts [42], in weaned sows [11], and in both gilts and sows [10]. Much of this information is limited and the methodology quite variable. Differences in the approach and effectiveness appear to result from alternative use of gonadotropins, such as eCG, to synchronize follicle development, use of induction hormone GnRH or one of its agonists (pLH or hCG), the dosage and time of administration for the induction hormone, and the time of AI following induction [15,22]. Another GnRH analogue, lGnRH-III (peforelin, Maprelin^®^ XP10, Veyx-Pharma, Schwarzenborn, Germany) did not show significant differences in reproductive parameters and litter size when compared with untreated gilts., even though peforelin seemed to have a positive effect on follicle growth [11,43,44].

Only a few studies used the GnRH analogue buserelin in FTAI protocols for gilts. Martinat-Botté [19] used buserelin as to effectively stimulate LH secretion to induce ovulation in gilts, followed by applying a double FTAI-CAI protocol (3 × 10^9^ spermatozoa). Pregnancy, farrowing rate, and litter size were similar to our results from a single FTAI-PCAI buserelin protocol in gilts.

Most recently, the intravaginal application of a gel containing the GnRH analogue triptorelin (OvuGel^®^; Elanco, Guelph, ON, Canada) was promoted in both sows and gilts [1,22,45,46]. Use of triptorelin is recommended in weaned sows. They received 200 Ug of triptorelin either 96 h after weaning or at estrus detection and were subsequently inseminated with a double FTAI-PCAI [1,21] and a single FTAI-PCAI [15,47]. The use of 100–400 µg triptorelin with FTAI in gilts at 120 h after Regumate treatment obtained great results for ovulation induction, but the optimal timing of triptorelin administration after Regumate protocol might not yet have been identified in gilts [22]. In contrast, a single FTAI-CAI triptorelin protocol applied 120 h after Regumate treatment in gilts showed a farrowing rate lower than in the control group (80.3% versus 89.5%) [24]. Future research must be conducted using FTAI triptorelin protocols in gilts.

Estrus duration was shorter in gilts treated with buserelin *(p* < 0.001). Ulguim [15] did not find a significant effect of intramuscular pLH on estrus duration in gilts, but when pLH was administered by the vulvar submucosa route, estrus duration significantly decreased with respect to the control group (*p* < 0.05). Martinat-Botté [19] showed that double FTAI with buserelin in gilts can reduce estrus duration (*p* < 0.05). Driancourt [18] and Pearodwong [48] used buserelin in sows, and no significant modification was found in both estrus and weaning-AI interval duration. Similar results were found when using peforelin in gilts [43,44].

The proposed protocol, buserelin plus single FTAI-PCAI, is aimed at breeding synchronization through ovulation induction and the efficient use of semen doses with the PCAI technique. Therefore, farrowing would be expected to be grouped in a short period. The advantages of all sows farrowing close together includes improved labour efficiency in the farrowing room due to easier cross-fostering, better supervision of farrowing, and efficient processing of litters [15,24]. Our results showed a significant effect of season on delivery batch duration, with shorter values in autumn. 

The sow has an important physiological basis inherited from the reproductive seasonality pattern of its ancestor, the wild boar. The European wild boar (Sus scrofa) is considered a short day breeder due to its circadian secretion pattern of melatonin [49]; in this way, wild boar piglets are born in autumn, when food availability is high. Management factors (temperature, light, and feed) control the functioning of the hypothalamic-hypophysis-ovarian (HHO) axis and select autumn as the best time of year for reproduction. During summer and early autumn, the reproductive seasonality syndrome is frequent; this syndrome is caused by an imbalance of the HHO axis that affects follicular development and results in decreased reproductive parameters and increased percentage of anestrus gilts in [50].

Usually, female wild boars have ovarian activity only from November to April [51]. This shared pattern with its domestic descendant [52] would be the cause of the shorter delivery batch duration observed in autumn. Until now, negative seasonal effects in the pig industry included delayed puberty in gilts, prolonged weaning-to-estrus interval, reduced farrowing rate, and reduced litter size [53], but we did not find any reference to seasonal effects on delivery batch duration. On the other hand, under buserelin action, the delivery batch duration in spring was similar to that of autumn [49,54,55].

Seasonal effects on sperm characteristics may also play a role. Seasonal influence on protamine-like proteins has been demonstrate in invertebrates (*Myrtilus galloprovinciallis*); these proteins are the major basic component of sperm chromatin and several ambient factors (including pollutants) can modify their ability for binding and protecting DNA, so that their alteration allows sperm DNA fragmentation, causing infertility [56,57].

Several studies in mammals showed similar results. In humans, seasonal changes in sperm count and chromatin condensation were described with maximum values in January and April, respectively; no circannual relation was observed for motility and vitality [58]. However, circannual variation of morphology and motility of human semen were more recently reported; the rate of sperm with fast forward motility decreased from spring to autumn, with a recovery in winter, while the percentage of sperms with normal morphology was significantly higher in spring when compared with summer [59]. Seasonal effects on the chromatin status were also observed in semen from ovine, Iberian red deer and brown bear; in the breeding season, chromatin was less condensed and this status may be related to enhanced spermatogenesis [60]. In stallion semen, elevated DNA fragmentation and both low sperm motility and viability were detected in midsummer [61].

In boar, distinct motile sperm subpopulations occur in extended semen, and their proportions vary according to the season of collection; summer and autumn seem negative impact on the fast and linear subpopulation [62]. Additionally in boar, a reduction in sperm concentration was detected in spring and summer, although most seminal parameters were constant year-round [63]. As recently showed [64], DNA fragmentation was significantly higher when semen was produced in the increasing photoperiod respect to the decreasing photoperiod; the lowest values corresponded to autumn, while the highest values were found in summer.

Ambient pollutants can also play an important role in male fertility. In humans, metal pollutants as copper and chromium altered protamines/histones ratio and DNA binding model; therefore, the protective action of these proteins was reversed and they were involved in DNA oxidative damage, resulting in a higher DNA fragmentation index in the spermatozoa [65]. The effect of these metal pollutants could be transgenerational [66] Boar used in this study belonged to an insemination center, located in the countryside, and no industrial pollution was reported in its vicinity. Also, no nutritional problems affecting sperm production occurred in this center.

FTAI protocols including the use of GnRH analogues are aimed at improving piglets’ weight at birth (individual and litter weight), as well as litter weight homogeneity [67]. Our protocol resulted in increased individual weight at birth, but no differences were found for litter weight and litter homogeneity. As can be seen in Table 5, mean piglet weight from C and T groups differed in only 0.023 kg (23 g). The sample sizes were large in both groups and the SE values were low; in these conditions, the power of statistical test is high and therefore very small differences may appear as statistically significant. A different issue is the biological significance of this very small difference detected as statistically significant; it only accounts for a 1.74% increase in weight. Furthermore, the resolution was 20 g for the used scale, very near the detected difference between means. The resolution of a scale is the smallest readable difference between two measured values. On the other hand, delivery was not induced in this farm and no significant differences were detected between groups for gestation length; gestation length would not explain for the detected difference in birth weight. Hence, the applied hormonal treatment can be assumed not to worsen the newborn piglet’s weight. No significant differences were found between groups for total piglets born/litter, and therefore, the slightly increased individual weight at birth in the T group was not reflected in the litter weight. Litter homogeneity was high in both the T and C groups; good management on the studied farm could explain this finding. Vangroenweghe [44] reported that treatment with another GnRH analogue (peforelin) did not result in a significant increase in individual weight at birth or litter weight homogeneity. 

The proposed protocol implies the use of an additional treatment (buserelin) with respect to the usual protocol (double PCAI). However, since only one insemination is needed, the cost of semen, material, and personnel is reduced; therefore, the proposed protocol represents a 36% reduction in the cost of the usual protocol. Furthermore, the proposed protocol shows other advantages shown below [68]. Synchronizing ovulation and reducing the number of inseminations allow a better organization of farm work. Additionally, greater grouping of deliveries becomes possible, allowing better care of mothers and newborn piglets. Using a single dose of semen allows selected boars to be tested on a larger number of females in less time, so their progeny tests can be completed more quickly. In this way, a greater speed in the diffusion of genetic improvement is achieved in production farms. All these advantages are achieved without worsening the productive and reproductive indices, as shown in the present work.

## 5. Conclusions

The present study proposes, as a novelty, the combination in gilts of single FTAI plus buserelin with the PCAI technique. Only a small percentage of unsuccessful probe passage was found (10.2%; 23/226). The frequency of semen backflow, metritis, and bleeding was also small (global frequency: 14.8%; 30/203). There were no significant impacts on reproductive, prolificacy, and both gestation and weaning-AI interval duration parameters. Significant decreases in both estrus and delivery batch duration in spring were detected. Additionally, a significant increase in individual birth weight was found. According to our results, the use of the single FTAI-PCAI with buserelin is recommended in gilts. Using this new combined technique reduces both seminal doses and number of AIs; therefore, an important optimization of boars and semen doses is achieved. In this way, each gilt only needs 1500 × 10^6^ spermatozoa (one dose) to get pregnant, versus the 6000 × 10^6^ spermatozoa (two doses) needed with the usual technique.

## Figures and Tables

**Table 1:** Problem frequencies for every inseminated gilt. Data are reported as percentages and, in brackets, count/*n*, where *n*: number of gilts.

Group	AI1		AI2	
	Problem	% (Count/*n*)	Problem	% (Count/*n*)
C	None	77.5 (186/240)	None	78.8 (189/240)
	Unsuccessful probe passage	13.3 (32/240)	Unsuccessful probe passage	10.5 (25/240)
	Difficult probe passage	6.2 (15/240)	Difficult probe passage	9.1 (22/240)
	Semen backflow	1.3 (3/240)	Semen back flow	0.4 (1/240)
	Metritis	0.4 (1/240)	Metritis	0.8 (2/240)
	Bleeding	1.3 (3/240)	Bleeding	0.4 (1/240)
T	None	76.5 (173/226)		
	Difficult probe passage	11.5 (26/226)		
	Unsuccessful probe passage	10.2 (23/226)		
	Metritis	0.9 (2/226)		
	Bleeding	0.4 (1/226)		
	Metritis and bleeding	0.4 (1/226)		

**Table 2:** Reproductive and production performances for studied gilts. Data are reported as percentages and count/*n* (for pregnancy rate, farrowing rate (where *n*: number of gilts), and dystocia (where *n*: number of deliveries); mean ± SE (standard error) is shown for total piglets born/litter, live-born piglets/litter, stillborn piglets/litter, and mummies/litter.

Variable	C	T	*p*-Value
	(*n* = 194 Gilts)	(*n* = 203 Gilts)	
Pregnancy rate	92.3 (179/194)	94.1 (191/203)	0.602
Farrowing rate	89.2(173/194)	91.1 (185/203)	0.627
Dystocia	19.1 (33/173)	20.0 (37/185)	0.931
Total piglets born/litter	18.52 ± 0.291	18.12 ± 0.284	0.339
Live-born piglets/litter	17.38 ± 0.274	17.04 ± 0.277	0.984
Stillborn piglets/litter	0.90 ± 0.179	0.80 ± 0.108	0.356
Mummies/litter	0.23 ± 0.041	0.31 ± 0.052	0.396

No significant differences were found in any case (*p* > 0.05).

**Table 3:** Estrus, gestation, and weaning-AI interval duration for the studied gilts. *n*: number of gilts.

Variable	C (*n* = 194 Gilts)		T (*n* = 203 Gilts)		*p*-Value
	Mean ± SE	Median ± SE	Mean ± SE	Median ± SE	
Estrus duration (h)	62.613 ± 0.235	64.000 ± 0.103	59.542 ± 0.301	59.000 ± 1.039	<0.001
Gestation duration (d)	113.133 ± 0.470	115.000 ± 0.231	114.595 ± 0.289	115.000 ± 0.150	0.303
Weaning-estrus (d)	6.089 ± 0.396	5.000 ± 0.149	7.119 ± 0.406	5.000 ± 0.133	0.787

Significant differences between groups were only detected for estrus duration, which was significantly shorter in the T group (*p* < 0.001). According to median values, 50% of gilts in the T group finished estrus in 59 h, versus 64 h for the C group.

**Table 4:** Delivery batch duration of studied gilts in total, T, and C groups. SE: standard error, *n*: number of deliveries.

Season	Total			C			T			*p*-Value
	*n*	Mean ± SE	Median ± SE	*n*	Mean ± SE	Median ± SE	*n*	Mean ± SE	Median ± SE	
Spring	112	2.830 ± 0.100 ^A^	3.00 ± 0.168	57	3.053 ± 0.143 ^b^	3.00 ± 0.247	55	2.600 ± 0.134 ^a^	2.00 ± 0.189	0.018
Summer	29	3.448 ± 0.202 ^B^	4.00 ± 0.226 ^B^	14	3.500 ± 0.327	3.00 ± 0.624	15	3.400 ± 0.254	4.00 ± 0.161	0.856
Autumn	216	2.546 ± 0.079 ^C^	2.00 ± 0.088	101	2.5045 ± 0.127	2.00 ± 0.173	115	2.548 ± 0.098	2.00 ± 0.102	0.747

^a,b^: different letters in the same row mean significant differences (*p* < 0.05). ^A,B,C^: different letters in the same column mean significant differences (*p* < 0.05).

**Table 5:** Piglets’ weight within 24 h after birth and before cross-fostering (individual and litter weight, CV: coefficient of variation). SE: standard error, *n*: number of data.

Variable	T		C		*p*-Value
	*n*	Mean± SE	*n*	Mean± SE	
Individual weight (kg)	2893	1.3429 ± 0.00329	2725	1.3199 ± 0.00298	<0.001
Litter weight (kg)	169	22.9294 ± 0.42655	158	22.7570 ± 0.42846	0.186
CV	169	6.7141 ± 0.41948	158	7.5326 ± 0.38916	0.092

## Data Availability

Data supporting reported results can be sent to anyone interested by contacting the corresponding author.
